# Application of exogenous salicylic acid reduces disease severity of *Plasmodiophora brassicae* in pakchoi (*Brassica campestris* ssp. *chinensis* Makino)

**DOI:** 10.1371/journal.pone.0248648

**Published:** 2021-06-24

**Authors:** Dandan Xi, Xiaofeng Li, Lu Gao, Zhaohui Zhang, Yuying Zhu, Hongfang Zhu

**Affiliations:** 1 Horticultural Research Institute, Shanghai Academy of Agricultural Sciences, Shanghai, China; 2 Shanghai Key Lab of Protected Horticultural Technology, Shanghai, China; 3 Zhuanghang Comprehensive Experiment Station, Shanghai Academy of Agricultural Sciences, Shanghai, China; Huazhong Agriculture University, CHINA

## Abstract

Clubroot is one of the most serious diseases affecting *Brassicaceae* plants worldwide. However, there is no effective control method for clubroot. Salicylic acid (SA) is a plant hormone that plays a critical role in plant defense. In our study, we found the disease severity of a clubroot-sensitive cultivar of pakchoi, Xinxiaqing, was reduced with 0.6mM exogenous SA after the infection of *P*. *brassicae*. To investigate the mechanism of SA-reduced disease severity against clubroot, then we analyzed the plant growth, alteration of antioxidant enzyme system, and related gene expression of Xinxiaqing. Results showed that the clubroot incidence rate and disease index were decreased after being treated with 0.6 mM exogenous SA. Furthermore, plant growth, reactive oxygen species (ROS) contents, and membrane lipid peroxidation were changed. The activities of antioxidant enzymes, including superoxide dismutase (SOD), ascorbic acid-peroxidase (APX), catalase (CAT), and glutathione reductase (GR), were increased. Additionally, the production rates of malondialdehyde (MDA), hydrogen peroxide (H_2_O_2_), and superoxide anion (O_2_^·–^) were also inhibited. The expression levels of genes, encoding *SOD*, *APX*, *CAT*, and *GR*, were increased. By summering all results, we conclude that 0.6 mM SA contributes to the reduction of disease severity to clubroot by increasing the activities of antioxidant enzymes, abilities of osmotic regulation, and ROS scavenging to reduce the clubroot-induced damage in pakchoi.

## Introduction

Clubroot is a worldwide soil-borne disease that is caused by *Plasmodiophora brassicae* Woron (*P*. *brassicae*) infection [1]. *P*. *brassicae* can infect over 100 *Brassica* species, including *Brassica napus* L., *Brassica rapa pekinesis*, *Brassica oleracea* L., and *Brassica rapa* L. *P*. *brassicae* infection often causes abnormal cell enlargement and division in roots, which affects water and nutrient transport, resulting in reduced crop production and great economic loss. In recent years, damage of clubroot becomes more and more severe. According to the statistics, the clubroot incident area had already reached to 4666.7 hm^2^ in Shanghai (China) in 2017 (supplied by Shanghai Agricultural Technology Extension Service Center). The life cycle of *P*. *brassicae* is mainly composed of the primary phase, taking place in root hairs, and the secondary phase, taking place in cortical and stele cells [2,3]. Therefore, many researchers pay much attention to control clubroot, including chemical control, crop rotation, and genetic control [1].

To overcome pathogen infections, plants have developed a defense system comprising of pathogen-associated molecular patterns (PAMP)-triggered immunity (PTI) and resistance (R) proteins associated with pathogen effector-triggered immunity (ETI) [4,5]. Following activated PTI or ETI, the mobile signals, produced in local infected tissues, move to distal tissues, activating systemic acquired resistance (SAR) [5]. SA is a small phenolic molecule in plants and well-studied as a signal molecular during plant immunity response, although it also plays essential roles in cellular signal transduction, plant growth and development, and abiotic stresses [6–8].

Oxidative stress acts as a result of various stresses, including biotic stresses. The primary reason for oxidative stress is the excessive accumulation of reactive oxygen species (ROS) that is produced during aerobic metabolism and damaged plant cells [9,10]. To avoid ROS damage, plants have evolved two systems comprising of non-enzymatic and enzymatic ROS scavenging mechanisms [9]. The involved enzymes mainly include superoxide dismutase (SOD), ascorbic acid-peroxidase (APX), glutathione peroxidase (GPX), catalase (CAT), and glutathione reductase (GR) [9]. Overproduction of ROS, beyond plant scavenging ability, further results in plant hypersensitive reaction (HR) and damages plant cells. Ozone (O_3_) and superoxide anion (O_2_^·–^) were reported to be accumulated in an *Arabidopsis* mutant, *radical-induced cell death 1* (*rcd1*), resulting in cell death [11]. Contrarily, overexpression of chloroplast SOD in chloroplast increased plant resistance to oxidative stress [12].

Plentiful evidence has revealed that SA is an effective strategy to inhibit clubroot disease. It has been hypothesized that SA can be converted to biologically inactive methyl salicylate by *P*. *brassicae* [13]. Exogenous SA was reported to suppress clubroot in *Arabidopsis* [14]. Similar results were also observed in broccoli (*B*. *oleracea)*, *Arabidopsis* Bur-0 accession [2,15]. Additionally, SA related genes were differentially expressed in *B*.*rapa* ssp. *chinensis* infected with *P*. *brassicae* [16]. However, the mechanism of SA repressing clubroot remains unclear.

Research has established that exogenous SA induced resistance to abiotic and biotic stresses, possibly through regulating ROS metabolism. Plants treated with SA increased APX and GR activity against temperature stress [17]. Application of benzo (1,2,3)thiadiazole S-methyl ester (BTH), an SA analog, induced wheat defense-related genes overexpression in wheat [18]. Moreover, SA application induced PR-proteins in *Solanum melongena L*. against *Verticillium dahlia* Kleb [19]. SA was also found to induce rubber tree against *Phytophthora palmivora* through increasing CAT, peroxidase (POD), and phenylalanine ammonialyase (PAL) activities [20]. A study revealed that the SA application enhanced two tomato cultivars’ resistance to the *Tomato yellow leaf curl virus* (TYLCV) by increasing POD and APX activities [21]. Recent research showed that exogenous SA decreased clubroot symptoms in two *Arabidopsis* accessions [2]. Conversely, degradation of endogenous SA enhanced *Ralstonia solanacearum* virulence in tobacco [22]. All these researches suggested that SA-mediated increased clubroot resistance may depend on the ROS pathway in pakchoi.

At present, there are a few pakchoi cultivars resistant to clubroot, and most cultivars exhibit susceptibility to clubroot, which brings challenges to growers and breeders[23]. Our previous researches have revealed 0.6 mM SA has the best effectivity to control clubroot [24]. However, the underlying molecular mechanism was not clear. In this study, we found that pakchoi exhibited increased clubroot resistance after exogenous SA, consistent with the previous result [24]. Additionally, ROS contents, antioxidant enzyme activities, and related gene expression were altered after SA treatment. We demonstrated that exogenous SA treatment increased antioxidant enzyme activities to scavenge over-produced ROS levels, resulting in increased clubroot resistance.

## Materials and methods

### Plant materials

Xinxiaqing, a pakchoi cultivar and mainly growing in Shanghai, was used for analysis and supplied by Protected Horticultural Research Institute, Shanghai Academy of Agricultural Science (SAAS). The *P*. *brassicae* (physiological race 7) was isolated from infected roots grew in Hongyang Farm in Qingpu District, Shanghai [23].

### *P*. *brassicae* and SA treatment

*P*. *brassicae* infected pakchoi roots with galls were harvested in fields and stored at -20°C until use. To extract resting spores, infected roots were washed with water and then grounded. The spore suspension was adjusted to 2×108 spores per milliliter and then mixed with soil (1000ml suspension/kg soil). pakchoi seeds were sowed on soil or mixed soil with spore suspension in a growth chamber, which was set to 28±1°C in light/20±1°C in dark with 12 hours light and 12 hours dark per day. The water content of the two types of soil was 60%. SA (Shanghai yuanye Bio-Technology Co., Ltd., China) was prepared in ethanol and then adjusted to 0.6 mmol/L by water. Ten days after germination (DAG), seedlings were treated with 0.6 mM SA or equal percentage ethanol dissolved in water (negative control) by application directly to the soil one time every day for four days. About 200 plants were used in each group. At forty DAG, plants were used to calculate disease index and disease incidence rate. The third leaves and whole roots were collected for RNA extraction, ROS content measurement, soluble protein content measurement, proline content measurement, and antioxidant enzyme activities.

### Disease assessment

Disease symptoms were discovered at forty DAG. The grade of incidence was described previously [25]. Grade 0: no symptoms; Grade 1: very small galls in lateral roots or primary roots, which do not affect main root growth; Grade 2: a few galls are found obviously in primary roots, and gall size is 2–3 times than transaction of the basal part of the stem; Grade 3: severe galls are found in lateral roots and primary roots, and gall size is over 4 times than transaction of the basal part of the stem, which affects plant growth [25].

Disease incidence rate (DIR) = (number of disease plants/total number of plants) X 100%

Disease index (DI) = ∑(disease grade X number of disease plants per grade) X 100 / (total number of plants)

Induced resistance index (IRI) = {(DI of inoculated plants- DI of inoculated plants with exogenous SA) / DI of inoculated plants} X100%。

### Measurement of proline content and soluble protein

Proline content was measured based on the method reported by Funck et al. [26]. Proline was extracted with sulfosalicylic acid, followed by acidic ninhydrin reagent. The mixture was extracted by toluene. The toluene supernatant was used to detect A_520_. Standard curves between 0 to 10 mM proline were treated in the same way to calculate proline content. Soluble protein content was detected using Coomassie Brilliant Blue G-250 [27].

### Measurement of ROS content

Malondialdehyde (MDA) content represents the damage of ROS to membrane lipids, and it was measured with the thiobarbituric acid (TBA) method [28]. H_2_O_2_ content was measured by monitoring the absorbance of titanium–peroxide complex at 415nm described by [29]. The O_2_^·–^content was measured by using the method described by Jang and [28]. In the presence of O_2_^·–^, nitrite is synthesized from hydroxylamine. A standard curve of NO2—was used to calculate O_2_^·–^content.

### Measurement of antioxidant enzyme activities

SOD activity was measured with nitro blue tetrazolium (NBT) according to the method used by Jiang and Zhang [28]. One-unit SOD activity was determined to cause 50% inhibition of the reduction of NBT at 560 nm. CAT and APX activity was measured by the method reported previously [28]. The reaction buffer, containing 50 mM potassium phosphate buffer (pH7.0), 10 mM H_2_O_2_, and enzyme extract, was used to detect the absorbance at 240 nm for measuring CAT activity. APX enzyme extract was added to reaction buffer containing 50mM potassium phosphate buffer (pH7.0), 0.5 mM ascorbate (ASC), 0.1 mM H_2_O_2_. The decrease of APX activity in A_290_ for 1 min was measured in the mixed solution at 25°C. GR activity was measured with the oxidation of NADPH at 340 nm [28]. The absorbance at 340 nm without NADPH was used as reference.

### RNA extraction and gene expression

To detect expression levels of *SOD*, *CAT*, *APX*, and *GR*, total RNA was extracted with RNAiso plus (Takara). Then cDNA was synthesized with HiScript II Q RT SuperMix for qPCR (+gDNA wiper) (Vazyme), after which qRT-PCR was performed with QuantStudio® 5 Real-Time PCR Instrument (96-well 0.2 ml Block) (Thermo Fisher Scientific). The following cycling conditions were used: 95°C 30s, 95°C 5s, 60°C 15s, and 72°C for 15s with a total of 40 cycles and 20μl reaction volume. Three technical replicates and biological replicates were used. Relative gene expression level was calculated by 2^-ΔΔCt^ method [30]. Actin was used as a reference gene. All primers used were listed in S1 Table.

#### Ethical approval

This article does not contain any studies with human participants or animals performed by any of the authors.

## Results

### The effects of SA on induced-clubroot-resistance and growth of pakchoi

**Fig 1:**
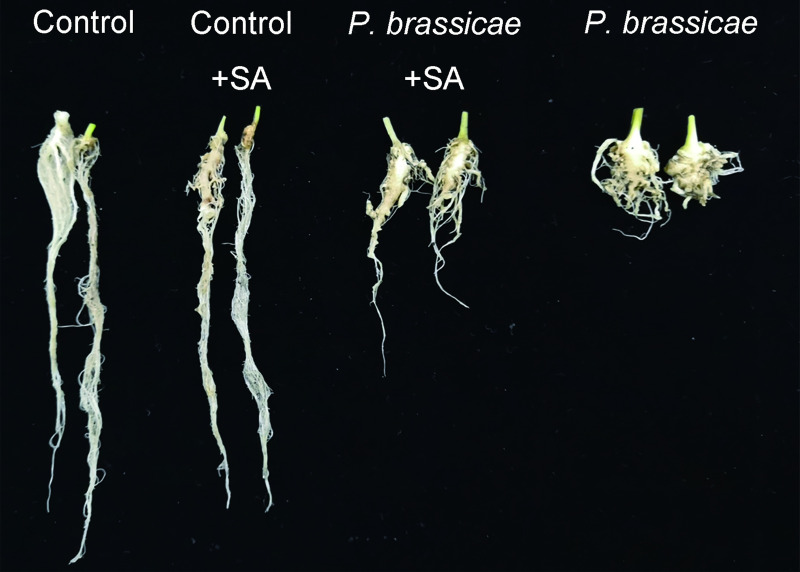
Forty-day old plant roots. Control and Control+SA: Uninoculated plants treated with ethanol or 0.6 mM SA at forty-day-old; respectively. *P*. *brassicae* and *P*. *brassicae* +SA: Plants sowed in mixed soil (with *P*. *brassicae*) treated with ethanol or 0.6 mM SA, respectively.

To explore whether SA increases pakchoi resistance to clubroot, seedlings having one leaf and one bud were treated with 0.6mM SA or an equal percentage of ethanol. There were no galls that occurred in roots of plants uninoculated with *P*. *brassicae* (Fig 1). Plant height, maximum leaf area, and fresh weight did not change with the SA application (Table 1). However, in plants inoculated with *P*. *brassicae*, an abnormal root system occurred with large galls before the SA application (Fig 1). DIR and DI of Group Inoculation were up to 66.15% and 36.16%, respectively (Table 1). Plant height, maximum leaf area, and fresh weight on the ground were reduced by 27.40%, 39.26%, and 58.61%, respectively, compared with those of the control group (Table 1). However, after 0.6 mM SA treatment for four days, DIR and DI were only up to 25.51% and 13.45% compared to those of the control group. DIR was reduced by 61.42% compared to that of inoculated plants, and DI was reduced by 62.80%. Additionally, plant height, maximum leaf area, and shoot fresh weight on the ground were only reduced by 17.22%, 19.42%, and 21.48%, respectively (Table 1). These data revealed that exogenous SA effectively released the inhibition of clubroot on pakchoi growth.

**Table 1:** Effect of SA on the induced-resistance and growth of pakchoi inoculated with *P*. *brassicae*.

Group	DIR(%)	DI(%)	IRI (%)	Plant height (cm)	Maximum leaf area (cm^2^)	Shoot fresh weight (g)
Control	-	-	-	13.65±0.51^a^	25.65±0.11 ^a^	6.33±0.07 ^a^
Inoculation	66.15±1.07^a^	36.16±2.03^a^	-	9.91±0.12 ^c^	15.58±0.13 ^c^	2.62±0.02 ^c^
Control+SA	-	-	-	13.30±0.41^a^	24.47±0.21^a^	6.21±0.03 ^a^
Inoculation+SA	25.51±2.43^b^	13.45±0.05^b^	61.21±0.017	11.30±0.46 ^b^	20.67±0.20 ^b^	4.97±0.11 ^b^

The calculation of DIR and DI was presented in the methods section. DIR: Disease incidence rate; DI: Disease index. Data shown represent mean ± SD (n = 200). The superscript letters indicate significance (Tukey’s HSD test, P < 0.05).

### Effects of SA on ROS, soluble protein, and proline contents of pakchoi

**Fig 2:**
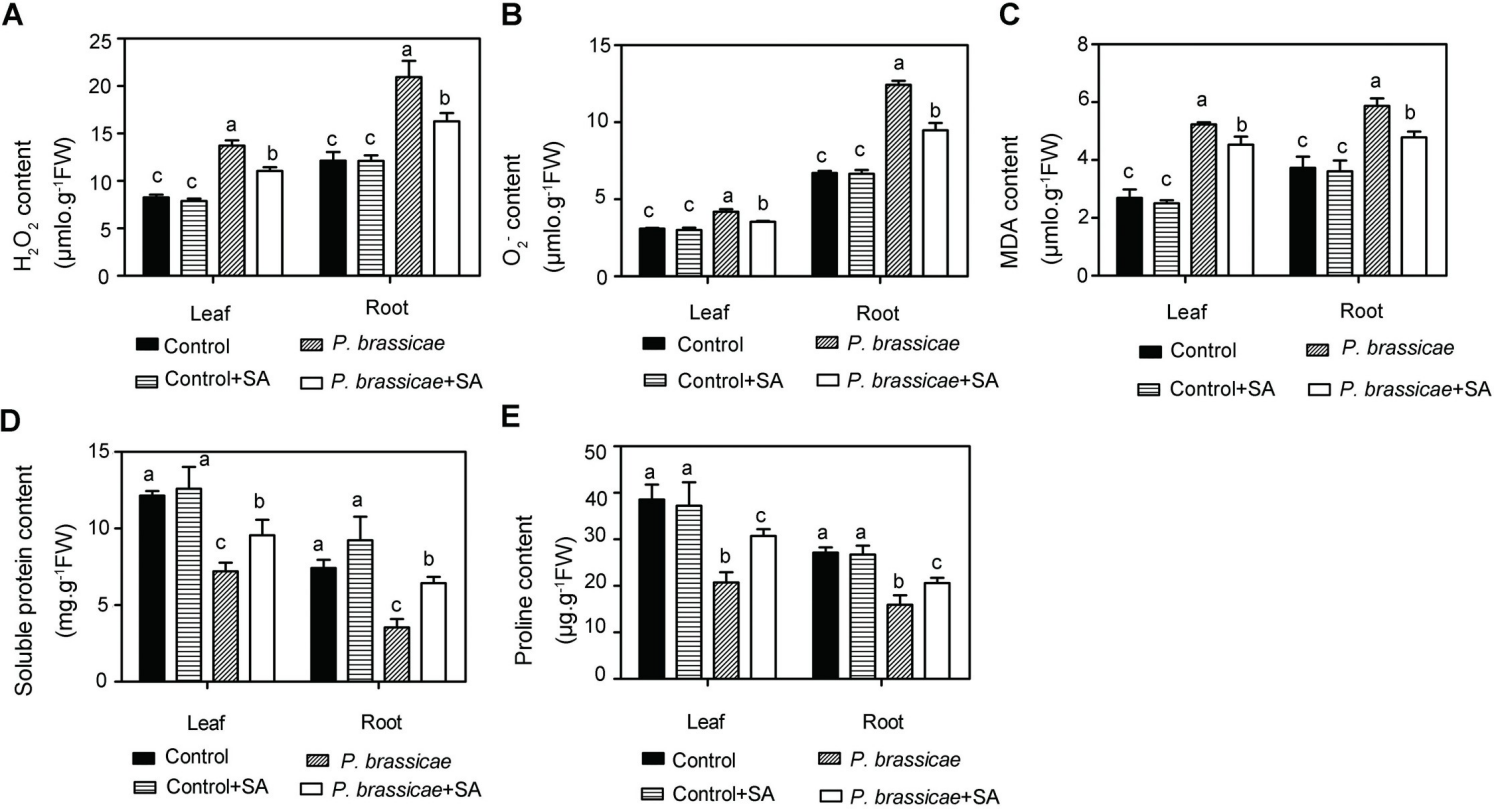
ROS, soluble protein, and proline contents in pakchoi. H_2_O_2_ **(A)**, O_2_^·–^**(B)**, MDA **(C)**, soluble protein **(D),** and proline contents **(E)** in leaves and in roots were presented. The third leaves and whole roots were harvested for analysis, respectively. Data shown represent mean ± SD (n = 3). Different letters indicate significance (Tukey’s HSD test, P < 0.05).

When suffering from biotic or abiotic stresses, plants will produce more ROS further leading to oxidative stress to cells. Accumulation of ROS causes cell membrane lipid peroxidation and cell death [10]. MDA is a byproduct of membrane lipid peroxidation and its content reflects the degree of membrane lipid peroxidation [10]. Firstly, we detected ROS expressed by H_2_O_2_ and O_2_^·–^contents in plants. In control plants, H_2_O_2_ contents were 8.25 μmol g^-1^ FW in leaves and 12.13 μmol g^-1^ FW in roots (Fig 2A). O_2_^·–^levels were 3.09 μmol g^-1^ FW in leaves and 6.71 μmol g^-1^ FW in roots (Fig 2B). MDA contents were 2.69 μmol g^-1^ FW in leaves and 3.73 μmol g^-1^ FW in roots (Fig 2C). However, ROS and MDA contents were distinctly increased in inoculated plants before SA treatment. H_2_O_2_, O_2_^·–^, and MDA contents were up to 13.72 μmol g^-1^ FW and 20.94 μmol g^-1^ FW, 4.20 μmol g^-1^ FW and 12.43 μmol g^-1^ FW, and 5.23 μmol g^-1^ FW and 5.87 μmol g^-1^ FW, in leaves and roots, respectively (Fig 2A–2C). After applying SA, H_2_O_2_ contents were reduced to 11.06 μmol g^-1^ FW and 16.28 μmol g^-1^ FW, in leaves and roots, respectively (Fig 2A–2C). Besides, O_2_^·–^of inoculated plants reduced to 3.54 μmol g^-1^ FW and 9.48 μmol g^-1^ FW while MDA reduced to 4.53μmol g^-1^ FW and 4.78 in leaves and in roots, respectively (Fig 2A–2C). These data revealed that *P*. *brassicae* inoculation significantly promoted ROS and MDA production and exogenous SA application inhibited ROS and MDA production to protect plants.

Plant resistance to stresses can be expressed by proline and soluble protein levels. Therefore, we detected the proline and soluble protein contents after the SA treatment. Soluble protein contents of inoculated plants were 7.21 mg g^-1^ FW in leaves and 3.55 mg g^-1^ FW in roots (Fig 2C). Proline contents were 20.72 mg g^-1^ FW in leaves and 15.94 mg g^-1^ FW in roots (Fig 2D). After 0.6 mM SA treatment, both soluble protein and proline contents were increased. Soluble protein contents reached 9.56 mg g^-1^ FW in leaves and 6.43 mg g^-1^ FW in roots (Fig 2C). Proline contents were increased to 30.74 mg g^-1^ FW in leaves and 20.61 mg g^-1^ FW in roots (Fig 2D). These results revealed that soluble protein and proline contents were inhibited by *P*. *brassicae* and promoted by SA.

### Effects of SA on antioxidant enzymes activities

**Fig 3:**
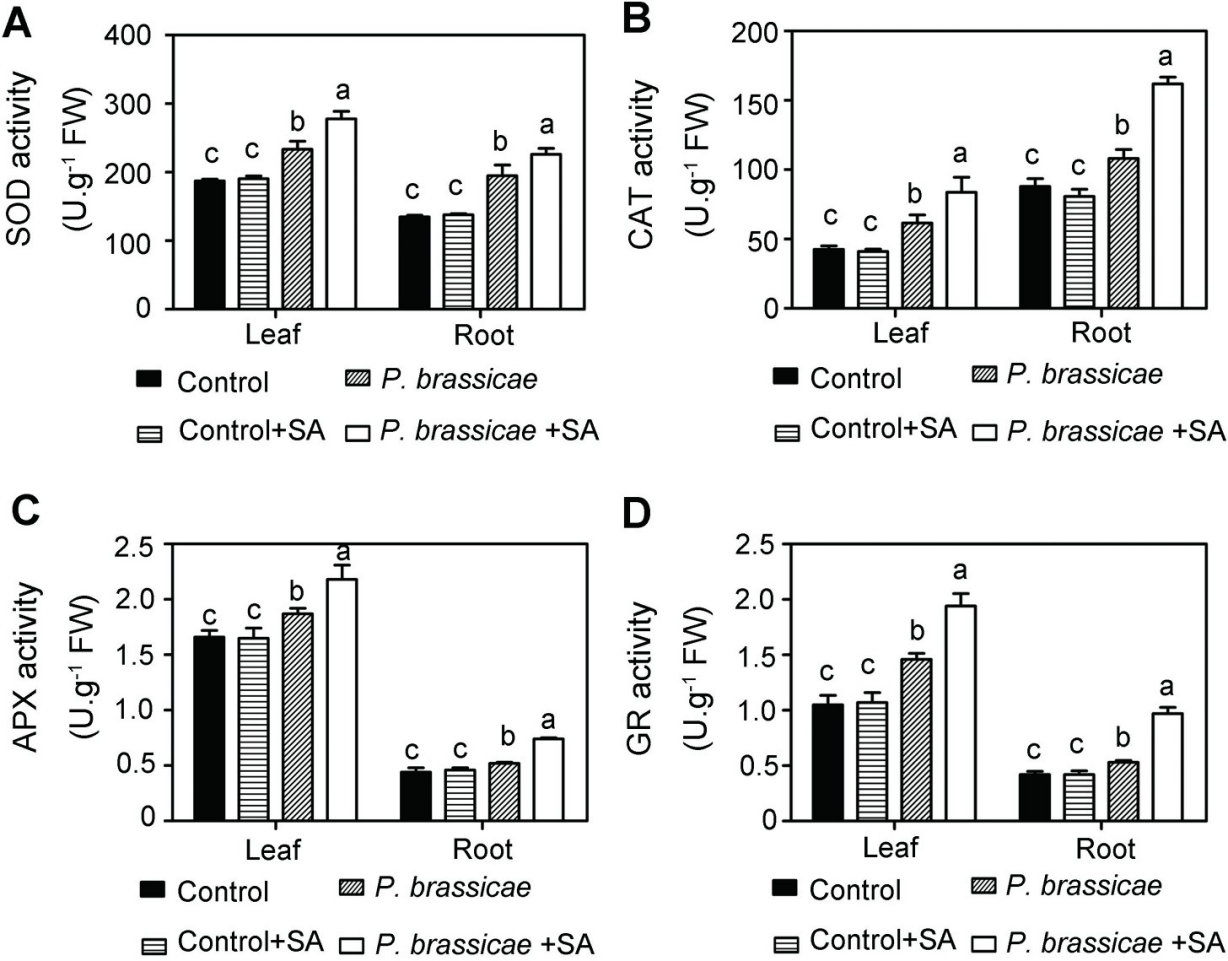
Antioxidant enzyme activities in pakchoi. SOD **(A)**, CAT **(B)**, APX **(C)**, and GR **(D)** activities in pakchoi were measured among four groups at 40 DAG. The data shown represent mean ± SD (n = 3). Different letters indicate significance (Tukey’s HSD test, P < 0.05).

To respond to stress, plants continuously adjust their metabolism to adapt to the environment, including increased activities of antioxidant enzymes to scavenge overproduced ROS. Among the uninoculated plants, SA treatment did not change SOD and GR activities but increased APX activities both in leaves and in roots (Fig 3). Among inoculated plants, SOD activities were 233.62 U g^-1^ FW in leaves and 194.74 U g^-1^ FW in roots, CAT activities were 61.53 U g^-1^ FW in leaves and 108.13 U g^-1^ FW in roots, APX was 1.66 U g^-1^ FW in leaves and 1.41 U g^-1^ FW in roots, and GR was 1.46 U g^-1^ FW in leaves and 0.53 U g^-1^ FW in roots (Fig 3). However, after 0.6 mM SA treatment of inoculated plants, SOD activities were up to 277.82 U g^-1^ FW in leaves and 226.09 U g^-1^FW in roots, CAT was up to 83.80 U g^-1^FW and 161.84 U g^-1^ FW, and GR was up to 1.94 U g^-1^ FW and 0.97 U g^-1^ FW, respectively (Fig 3). APX activities reached 1.95 U g^-1^ FW in leaves, but did not change in root compared to that of inoculating plants without SA (Fig 3). Increased antioxidant enzyme activities contribute to scavenging ROS and protect plants. These data revealed that SA induced plants resistance to clubroot through the antioxidant system.

### Effects of SA on antioxidant gene expression

**Fig 4:**
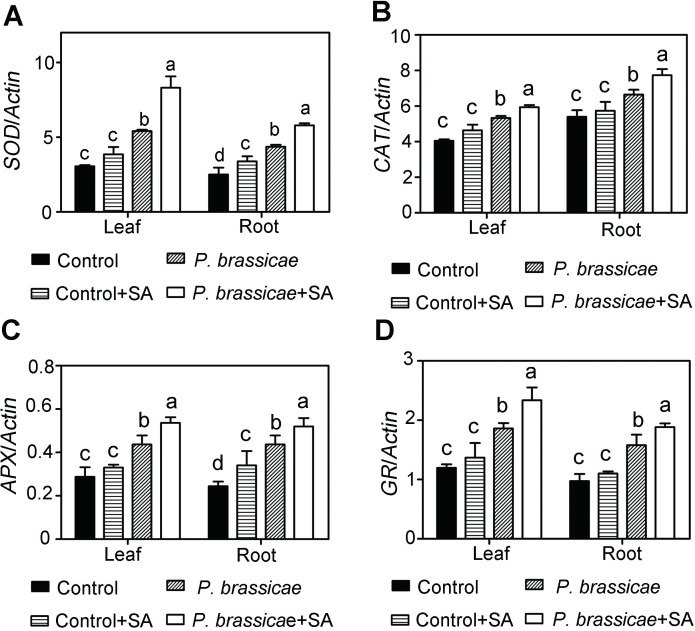
Relative gene expression levels in roots and leaves. SOD **(A)**, CAT **(B)**, APX **(C)**, and GR **(D)** expression levels in different groups were detected. The data shown represent the average ± SD (n = 3). Different letters represent significance (Tukey’S HSD test, P < 0.05).

Since activities of antioxidant enzymes in pakchoi were altered after inoculation and SA application, we further detected antioxidant gene expression levels to analyze the potential mechanism of SA–induced clubroot resistance in pakchoi. The qRT-PCR analysis revealed that exogenous SA did not alter *SOD*, *CAT*, *APX*, and *GR* expression levels both in leaves and in roots for the inoculated plants (Fig 4). When the inoculated plants were treated with SA, *SOD*, *CAT*, *APX*, and *GR* expression levels were increased significantly compared to uninoculated plants (Fig 4). These data revealed that exogenous SA promoted antioxidant enzyme biosynthesis through upregulating related gene expression, resulting in increased clubroot resistance.

## Discussion

SA, as a plant hormone, has been established to promote plant growth and development, inducing resistance to temperature and disease stresses. Exogenous SA has been verified to be beneficial in plant growth against abiotic and biotic stresses [31]. SA induces plant resistance to viral, fungal, and bacterial infections, which has been verified in tobacco and wheat [18,22]. In this study, inoculation with *P*. *brassicae* impacted plant height, maximum leaf area, and fresh weight. Our previous study revealed that different concentrations of exogenous SA treatment have different effects on clubroot incidence in pakchoi [24]. The clubroot incidence rate of plants treated with 0.6 mM SA was lower than that of plants treated with 0.2 mM, 0.4 mM, or 0.8 mM SA [24]. This suggests that 0.6 mM exogenous SA may work the best in controlling clubroot. Hence, we employed 0.6 mM exogenous SA, and after inoculation, it leads to decreased plant DIR, increased resistance to clubroot, and promoted plant growth (Fig 1 and Table 1).

Under normal conditions, the production and scavenging of cellular ROS keep dynamic balance. When plants suffer from stresses, the balance is broken and ROS is accumulated, resulted in a production rate is higher than the scavenging rate by antioxidant enzymes. Our study reveals that exogenous SA inhibits the production rate of H_2_O_2_ and O_2_^·–^, as well as MDA content. ROS scavenging mainly depends on antioxidant enzymes and enzyme activities. SOD, CAT, GR, and APX are key antioxidant enzymes required for ROS scavenging and preventing membrane from peroxidation. These four enzymes increase their activities after the SA treatment in plants inoculation (Fig 4). SOD converts O_2_^·–^into H_2_O_2_ [9]. The decreased O_2_^·–^might result from enhanced SOD activity induced by exogenous SA (Figs 3B and 4A). Moreover, SA treatment also inhibits the generation of H_2_O_2_ both in leaves and in roots, which is consistent with the enhancement of CAT and APX activities (Figs 3A, 4B and 4C). Ascorbic acid-glutathione (AsA-GSH) system comprises AsA, GR, and APX, converting H_2_O_2_ to H_2_O and MDA [9]. It has been established that SA can stimulate the AsA-GSH cycle to increase the efficiency of antioxidants. Our study revealed that APX and GR activities were increased, leading to decreased H_2_O_2_ and MDA contents (Figs 3A, 3C, 4C and 4D).

The secondary phase of *P*. *brassicae* lifecycle is associated with cell division and cell elongation, leading to gall formation [2]. Auxin is an essential hormone that plays a key role in root development, including gall formation [32]. Auxin level, as well as the expression of hormone-related genes, was changed after inoculation with *P*. *brassicae* in *B*.*napus* [33]. Previous research showed many pathogens positively regulate auxin biosynthesis or modulate auxin signaling to make hosts more susceptible to infection [34,35]. In *Arabidopsis*, it has been established that SA or its analog treatment represses auxin level and signaling [36]. Therefore, we hypothesize that the exogenous SA treatment may also affect auxin biosynthesis and signaling, therefore decreasing *P*. *brassicae* infection abilities and increasing plant resistance to infection, which is deserved to be further studied.

## Conclusions

In summary, we conclude that *P*. *brassicae*-infected pakchoi over products ROS and MDA, resulting in membrane lipid peroxidation, and damaging cell structures and functions. Exogenous SA significantly increases gene expression levels and antioxidant enzyme activities to decrease ROS contents and reduce or prevent membrane lipid peroxidation, leading to increased clubroot resistance in pakchoi.

## Supporting information

S1 Table Primers used for qRT-PCR. (XLSX)
